# Adaptation of Stability during Perturbed Walking in Parkinson’s Disease

**DOI:** 10.1038/s41598-017-18075-6

**Published:** 2017-12-19

**Authors:** Dario Martelli, Lan Luo, Jiyeon Kang, Un Jung Kang, Stanley Fahn, Sunil K. Agrawal

**Affiliations:** 1 Department of Mechanical Engineering, Columbia University, New York, NY USA; 2 Department of Neurology, College of Physicians and Surgeons, Columbia University, New York, NY USA; 3 Department of Rehabilitation and Regenerative Medicine, College of Physicians and Surgeons, Columbia University, New York, NY USA

## Abstract

Gait and balance disorders are major problems that contribute to falls among subjects with Parkinson’s disease (PD). Strengthening the compensatory responses through the use of balance perturbations may improve balance in PD. To date, it is unclear how PD affects the ability to react and adapt to perturbations delivered while walking. This study aims to investigate how PD affects the ability to walk, respond to balance perturbations, and produce acute short-term effects to improve compensatory reactions and gait stability. A cable-driven robot was used to train nine patients with PD and nine age-matched controls with multidirectional waist-pull perturbations while walking on a treadmill. Margin of stability and base of support were evaluated while walking without cables and reacting to the perturbations. PD was associated with a reduced stability in the forward direction and the inability to produce proactive anticipatory adjustments. Both groups were able to improve the response to the disturbances and produce short-term aftereffects of increased gait stability once the cables were removed. A single session of perturbation-based balance training produced acute effects that ameliorated gait instability in PD. This result is encouraging for designing new therapeutic interventions that remediate falls risk.

## Introduction

Parkinson’s disease (PD) is a neurodegenerative disorder that results primarily from a progressive loss of substantia nigra neurons within the basal ganglia that produce dopamine, an important neurotransmitter involved in the regulation of movement^1^. Individuals with PD commonly experience gait and balance disorders which contribute to falls, mobility loss, reduced independence and quality of life^2–4^. Balance impairment is difficult to treat with dopaminergic medication, which suggests a contribution by non-dopaminergic lesions^5^. In some cases, exercise and motor training can improve the performance of balance-related activities^3^. Nevertheless, there is no evidence that these improvements translate into prevention of falls^3^. This may be due to the fact that current training methods fail to target the specific neuromuscular skills required for fall prevention^6^.

With the aim to develop new methods that identify the causes of compromised balance in PD and to design specific pharmacological or training methods that help to reduce balance impairments, several groups have aimed to characterize and strengthen the control of the compensatory responses required after a balance perturbation^7–11^. The underlying hypothesis in these studies is that exposure to repeated perturbations can lead participants to better correct their loss of balance during the recovery phase (i.e., adaptation of the reactive strategy) and modify the volitional control of stability in face of a possible perturbation (i.e., adaptation of the proactive strategy). As a consequence, participants may show improved recovery after unexpected loss of balance encountered in daily life and, consequently, reduce their falls risk.

Generally, modifications of the neural, sensory and musculoskeletal systems in PD make the protective motor behaviors that counteract the lack of balance less effective, thus increasing the risk of falling^12–16^. However, potential for adaptation to motor training seems to be intact. By exposing patients to repeated controlled postural disturbances, the overall motor response against balance loss and their control of stability can be improved^7–11^. In the long term, the outcomes of multiple training sessions could be promising in terms of remediation of fall risk factors^17^ and reduction of falls^18–21^ in people with PD.

Despite the fact that most falls in PD are reported during walking^22^ and gait disorders are one of the hallmarks of PD^23–25^, the preceding literature is primarily focused on responses to balance perturbations delivered while standing. The role of the basal ganglia in motor learning, however, may be task dependent^26^.

**Figure 1:**
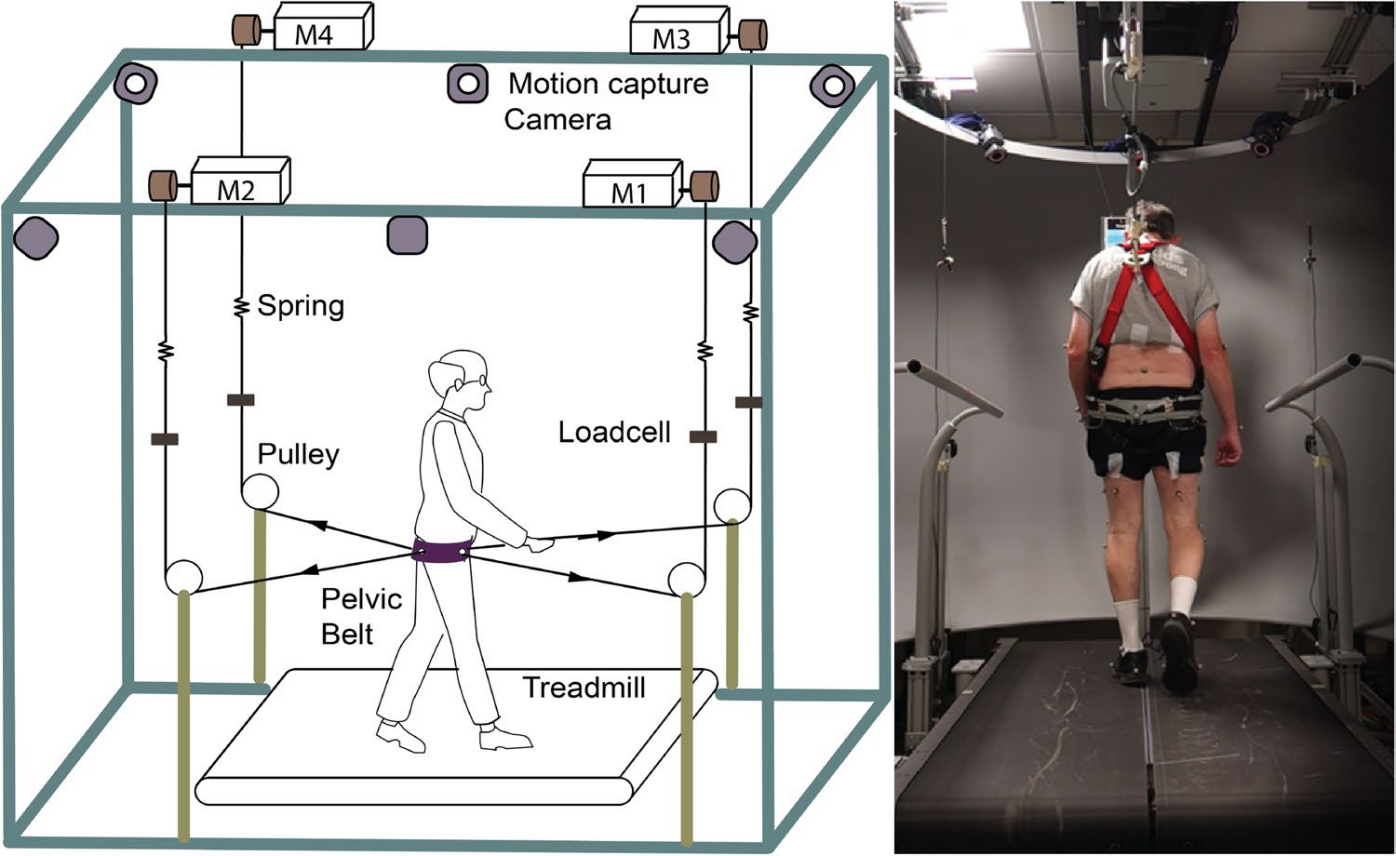
Experimental Setup. Schematic of the Active Tethered Assistive Pelvic Device (A-TPAD) and a picture of a participant walking with it. Four single-phase AC servo motors, powered in torque mode, are mounted on an inertial rigid frame and connected through low-stretch nylon-coated stainless steel wires and pulleys to a fabric hip belt worn by the subject. A load cell calibrated to record up to 890 N and a spring of stiffness 2.5 N/mm are installed in series with each cable to measure in real-time the instantaneous cable tension and reduce output impedance. A closed-loop controller ensures delivery of the correct tensions in the motors. Cables are routed using pulleys which are diagonally-directed. Waist-pull perturbations are provided by applying a transient force pulse on one or two of the four cables while walking at constant speed on a split-belt treadmill instrumented with two three-dimensional force plates (Bertec Instrumented Teadmill). A ten-camera motion capture system (Vicon Bonita-10 series) and force plates are used as a part of the controller. The motion capture system is used to track the cable attachment locations during a 30 seconds long calibration trial. The angle of each cable with respect to the fixed frame is recorded and averaged between steps for each time point of interest. In this way, a suitable cable tension value can be applied at each motor such that a resultant force of a desired amplitude proportional to the subject’s body weight (BW) is applied to the pelvis in the desired direction. The force plates are used to calculate the vertical Ground Reaction Forces (GRFs). This data is used to detect in real time gait events such as toe-off and heel strike (threshold at 50 N). These are used to time the application of perturbations in a repeatable and controlled way. When cables were attached to the subject, before giving the actual perturbation, a constant force of 25 N was applied by each motor to prevent cable slackening. The controller is implemented in Labview (National Instrument, PXI real time system).

Our group at Columbia University has developed the Active-Tethered Pelvic Assist Device (A-TPAD), an innovative cable-driven robot conceived for gait rehabilitation (Fig. 1)^27–29^. The A-TPAD consists of a lightweight belt worn by a participant on the pelvis to which several wires are attached. Desired forces and moments can be applied in any direction of the space and at precise time points in the gait cycle without adding inertia and rigid links to the human body. To date, it has been successfully used to improve gait in stroke survivors^30^ and in children with cerebral palsy^31^ by applying continuous forces at the waist-level. In this study, the A-TPAD was used to apply unpredictable force-controlled waist-pull perturbations for a short time duration. Previous experiments with healthy young subjects showed that a single session of perturbation-based balance training (PBT) was beneficial to improve gait stability^29,32^.

The aim of this study was to investigate to what extent PD affects the ability to walk, respond to balance perturbations, and produce acute short-term effects to improve compensatory reactions and control of unperturbed walking balance. We believe that the understanding of the mechanism of compensation and neuroplasticity to unexpected repeated perturbations during walking can fill an important gap in the current literature and have positive implications on the treatment of PD by helping to design effective training paradigms that remediate falls risk.

## Methods

### Participants

Nine subjects with idiopathic PD and nine healthy age-matched controls (HC) participated in the study. All experiments were performed in accordance with relevant guidelines and regulations. The experimental protocol was approved by the Institutional Review Board (IRB) of Columbia University. All subjects were informed about the research procedure and signed a written consent form approved by the IRB before participation.

Patients with PD were in the clinically ‘on’ state, approximately one hour after their last dose of dopaminergic medication. The severity of Parkinson’s disease was determined by a movement disorder specialist using the Hoehn and Yahr scale (H&Y)^33^ and the motor subscale of the International Parkinson and Movement Disorder Society-Sponsored revision of the Unified Parkinson’s Disease Rating Scale (MDS-UPDRS Part III)^34^. The average time over three trials to complete the Time Up and Go Test (TUG) and the 5-Meter Walk Test (5MWT) at participants’ comfortable walking speeds was used to assess ambulatory function.

### Experimental Setup and Protocol

Experimental sessions were carried out using the A-TPAD. Details on its design and control can be found in previous studies from our group^27–29^. Figure 1 shows the different components of the A-TPAD and a picture of a patient with PD walking on the treadmill with it.

**Figure 2:**
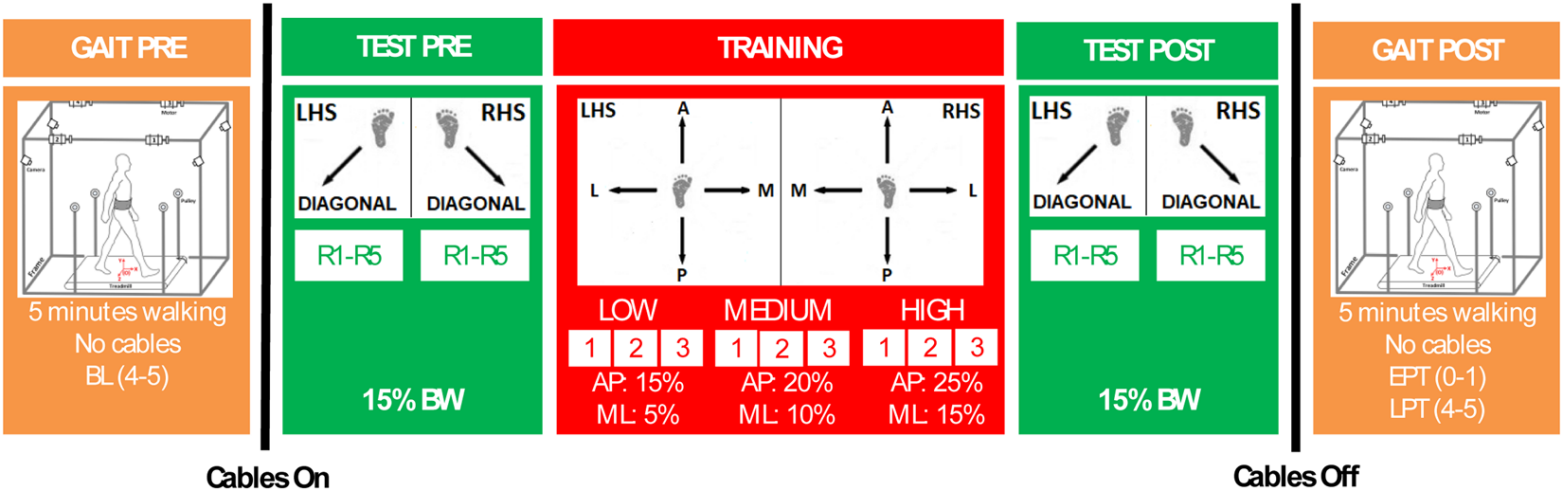
Experimental Protocol. Participants first walked on the treadmill for 5 minutes (GAIT PRE). Successively, the cables were attached to the brace. At the beginning, subjects were exposed to 10 posterior diagonal perturbations (TEST PRE). Perturbations consisted of 5 pulls with Motor 2 (back-right perturbation) triggered at right heel strike (RHS) and 5 pulls with Motor 4 (back-left perturbation) triggered at left heel strike (LHS) (Fig. 1, left panel). The first perturbation was delivered at RHS and then the order of perturbations was alternated. Peak force was fixed at 15% BW. Then, participants were trained with 9 blocks of 8 Antero-Posterior (AP) and Medio-Lateral (ML) perturbations of increasing intensities (TRAINING). 4 directions (Anterior – A; Posterior – P; Medial – M; Lateral – L), and 2 events (LHS; RHS) were used. At the beginning, the peak force was 15% BW and 5% BW for AP and ML perturbations, respectively. Every three blocks, the peak force was increased by 5% BW. The order of the perturbations in each block was chosen randomly. Time to complete each block was approximately 2 minutes. Before removing the cables, all subjects were exposed to the same set of diagonal perturbations delivered before the training session (TEST POST). Then, cables were removed, and all subjects walked for another 5 minutes (GAIT POST). Data collected during the last minute of GAIT PRE and the first and last minutes of GAIT POST were used in the analysis and were labeled as baseline, early and late post training (i.e., BL, EPT and LPT). All perturbations were delivered while walking at constant speed and consisted of a trapezoidal force profile (rise, hold and fall times of 150 ms duration each). The number of steps between perturbations was randomized (10–15 steps).

Figure 2 shows the experimental protocol. Participants first walked on the treadmill for 5 minutes (GAIT PRE). Successively, the cables were attached to the brace. At the beginning, subjects were exposed to 10 posterior diagonal perturbations with peak amplitude of 15% of the body weight (BW) (TEST PRE). Then, participants were trained with 9 blocks of 8 antero-posterior (AP) and medio-lateral (ML) perturbations of increasing intensities (TRAINING). Three amplitudes were used (low, medium, and high). They were equal to 15%, 20% and 25% for AP perturbations and 5%, 10%, and 15% BW for ML perturbations. The range of intensity of the perturbations was determined based on previous experiments with healthy young subjects^35^. Before removing the cables, all subjects were exposed to the same set of diagonal perturbations delivered before the training session (TEST POST). Then, cables were removed, and all subjects walked for another 5 minutes (GAIT POST). Data collected during the last minute of GAIT PRE and the first and last minutes of GAIT POST were used in the analysis and were labeled as baseline, early and late post training (BL, EPT and LPT). Participants were aware that they could be perturbed at the waist when the cables were attached, but were not informed about the magnitude, direction, or timing of the perturbations. Before the intervention started, they were instructed to try to maintain balance and keep walking.

Preferred walking speed was determined before the beginning of the experiment by gradually increasing the speed by 0.1 m/s until the subject reported that the speed was too fast and then reducing it by 0.1 m/s. Once determined, the walking speed was maintained constant during the experiment. In order to reduce the risk of fatigue, the treadmill was stopped every three blocks during the training session and the subjects were told that they could rest at any time if they felt fatigued. Over the duration of the experiment, subjects wore a safety harness that prevented them from falling, while not restricting their movements. The entire training session lasted approximately 30 minutes.

### Data Analysis

The trajectories of 55 reflective markers were collected at 200 Hz using a 10-camera motion capture system (Vicon Bonita-10 series). Marker placement was similar to reference^36^. The 3D marker trajectories were low-pass filtered with a cut-off frequency of 10 Hz using a fourth-order, zero-lag, Butterworth filter. Marker positions were used to define a 13-segment biomechanical model, similar to our previous studies^32,35–38^.

Heel strikes were determined when the vertical position of the lateral malleolus marker did not change by more than 2 mm in two consecutive time values^39^. To be consistent, all time events used in the analysis, including perturbation onset, were calculated using the kinematic data.

The effectiveness of the countermeasures to maintain balance are critically dependent on the individual’s ability to regulate the relationship between the body’s center of mass (CoM) and the base of support (BoS)^37,38^. The BoS in the AP/ML direction was estimated using the 4 markers attached on each foot (specifically calcaneus, tip of the toe, first and fifth metatarsals) as the difference between the most forward/lateral marker of the foot that completed the swing phase and the most backward/lateral marker of the contralateral foot. The AP and ML BoSs were used to quantify step length and step width, respectively. The 3D trajectory of the body’s CoM was calculated as the weighted sum of the 13 segments’ CoMs. Dynamic stability was determined using the margin of stability (MoS)^40^. The MoS in the AP/ML direction was quantified with respect to the last foot that completed the swing phase as:

1 $$MoS=Bo{S}_{Max}-XCoM$$

where BoS_Max_ is the anterior or lateral boundary of the BoS, estimated by the position of the tip of the toe or the fifth metatarsal in the AP and ML directions, respectively. The XCoM is the position of the extrapolated CoM and represents the state of the CoM taking into account both its position and velocity^41^. The AP and ML components of the XCoM were computed as:

2 $${\rm{X}}{\rm{C}}{\rm{o}}{{\rm{M}}}_{{\rm{A}}{\rm{P}},{\rm{M}}{\rm{L}}}={\rm{C}}{\rm{o}}{{\rm{M}}}_{{\rm{A}}{\rm{P}},{\rm{M}}{\rm{L}}}+{\rm{V}}{\rm{C}}{\rm{o}}{{\rm{M}}}_{{\rm{A}}{\rm{P}},{\rm{M}}{\rm{L}}}/\sqrt{{\rm{g}}/{{\rm{h}}}_{{\rm{C}}{\rm{o}}{\rm{M}}}}$$

where CoM_AP,ML_ and V*CoM*_*AP*,*ML *_are the AP or ML position and velocity of the CoM, h_CoM_ is the estimated pendulum length (instantaneous distance between the body CoM and the ankle joint of the leading leg) and g is the gravitational acceleration. The treadmill speed was added to the V*CoM*_*AP*_^42^. Noticeably, a larger MoS is associated with a higher stability.

During unperturbed gait, outcome measures were quantified by the average values calculated at the first 20 right heel strikes of BL, EPT and LPT. During the compensatory reaction, outcome measures were calculated during the perturbations delivered at right heel strike. Two time events were identified: perturbation onset (PON) and the compensatory heel strike (CHS – first heel strike after PON). MoS_PON_ and BoS_PON_ were used to identify pre-perturbation stability and possible proactive adaptations in the gait pattern. It must be noted that at PON the applied forces are still at zero. MoS_CHS_ and BoS_CHS_ were used to identify post-perturbation stability and possible reactive adaptations to the early compensatory reaction.

### Statistical Analysis

Potential differences in anthropometric measures (age, height, weight, and gender), ambulatory functions (time to complete the TUG and overground speed in the 5MWT) and preferred treadmill walking speed between the two groups were investigated by independent samples t-tests or Mann Whitney U Test, when appropriate. Data was analyzed by means of mixed design repeated-measures ANOVAs. During unperturbed gait, main and simple interaction effects of group (2 levels, PD and HC) and session (3 levels: BL, EPT and LPT) were analyzed. During training, main effect of group (2 levels, PD and HC) and simple interaction effects between group and direction (4 levels: Anterior, Posterior, Medial and Lateral), amplitude (3 levels: Low, Medium and High), and repetition (3 levels: R1-R3) were examined using the outcomes at CHS as dependent measures. During the test, main and simple interaction effects of group (2 levels, PD and HC), session (2 levels: PRE and POST), and repetition (5 levels: R1-R5) were investigated using the outcomes at PON and CHS as dependent measures. For all analysis, group was the only between-subject factor. If significant, main and interaction effects were followed up by pairwise comparisons with Bonferroni’s correction. The Lilliefors, Levene’s and Mauchly’s tests were performed to check the normality, homoscedasticity and sphericity assumptions of data. The Huynd-Feldt correction was applied if data violated the sphericity condition. Statistical significance was set at p < 0.05.

### Data Availability

The datasets analyzed during the current study are available from the corresponding author on request.

## Results

The two groups exhibited similar (*p* > 0.230) anthropometric measures, ambulatory function and preferred treadmill walking speed (Table 1). PD patients enrolled were in mild disease states: the H&Y stages ranged from 1 to 2^33^ and the MDS-UPDRS Part III scores ranged from 8 to 25^43^ (Table 1). After the perturbation, all participants were able to recover their balance without falling. All except two participants completed the experiment without difficulty: two PD subjects did not perform the third block of training due to fatigue.

**Table 1:** Participants Characteristics.

Variable	PD Group	HC Group	p-value
Age [years]	64.3 ± 7.4	64.7 ± 7.3	0.924
Gender [male/female]	7/2	6/3	1
Height [cm]	169.6 ± 6.1	172.9 ± 6.1	0.261
Weight [kg]	75.5 ± 15.7	75.6 ± 8.9	0.993
TUG [s]	9.46 ± 3.70	7.81 ± 1.44	0.230
5MWT [m/s]	1.39 ± 0.11	1.42 ± 0.15	0.606
Treadmill Speed [m/s]	0.89 ± 0.12	0.9 ± 0.17	0.871
H&Y [0–5]	1.78 ± 0.44	—	—
MDS UPDRS III [0–132]	14.44 ± 6.44	—	—

Baseline anthropometric and functional characteristics of the Parkinson’s Disease (PD) and Healthy Controls (HC) groups. Values are reported as mean ± one standard deviation. TUG: Time Up and Go test. 5MWT: Five Meters Walking Test. H&Y: Hoehn and Yahr staging of severity of Parkinson’s disease. MDS-UPDRS III: Movement Disorder Society Unified Parkinson’s Disease Rating Scale Part III score.

### Unperturbed Gait

**Figure 3:**
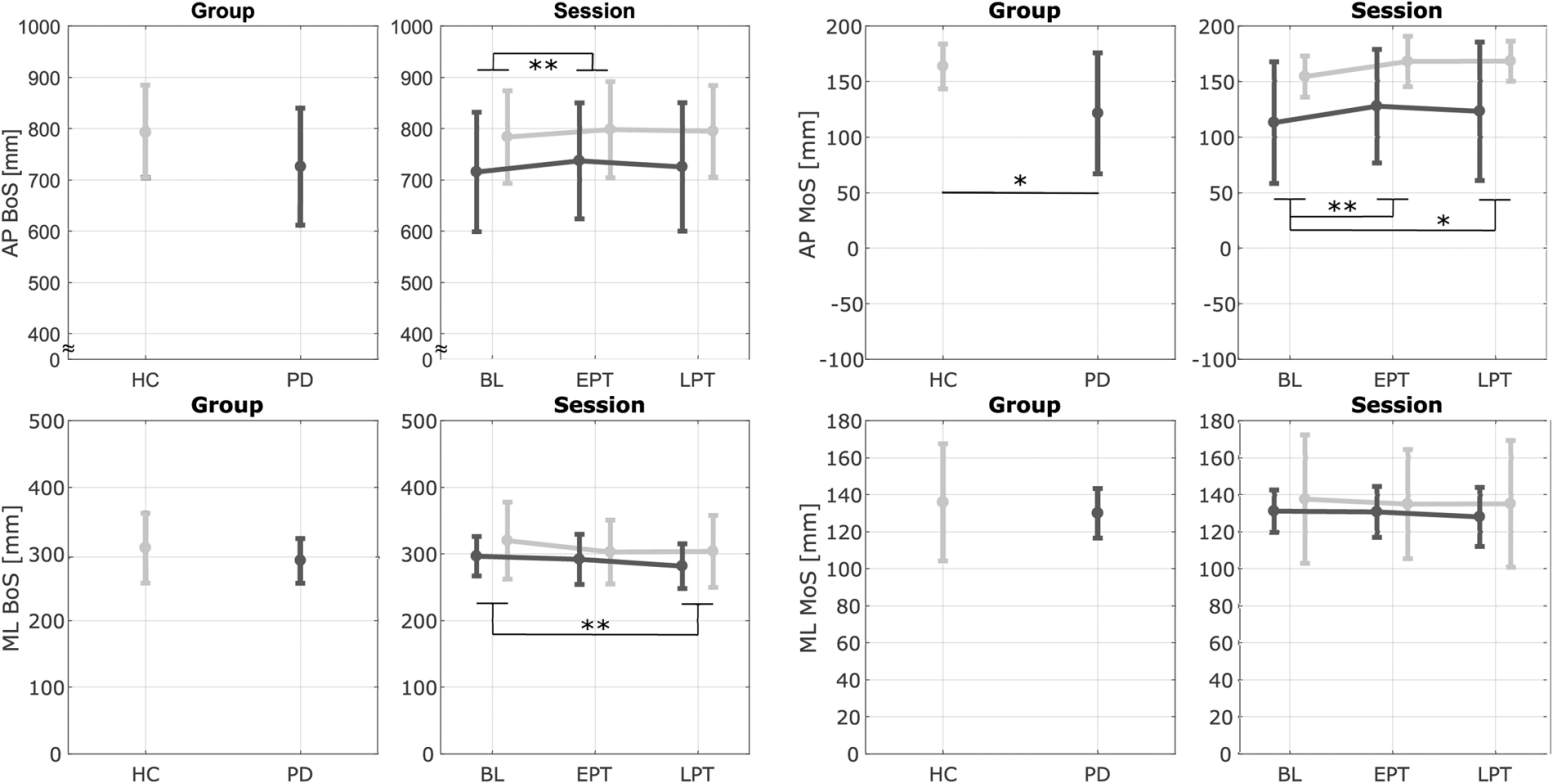
Results - Unperturbed Walking (without cables). Average Antero-Posterior (AP) and Medio-Lateral (ML) Margin of Stability (MoS) and Base of Support (BoS) during the first 20 right heel strikes of unperturbed walking for each group (Parkinson’s Disease - PD and Healthy Controls - HC) and session (Baseline - BL, Early Post Training - EPT and Late Post Training - LPT). Dark and light gray lines represent the PD and the HC groups, respectively. Bars refer to standard deviations. *Significant effect of the pairwise comparisons with Bonferroni’s correction (*p < 0.05, **p < 0.01, ***p < 0.001).

Figure 3 reports the results of unperturbed walking without cables (GAIT PRE & POST in Fig. 2). The AP MoS showed a significant main effect of group (*p* = 0.044), such that PD patients always walked with a lower AP MoS than HCs.

The exposure to repeated perturbations during training produced short-term aftereffects such that when cables were removed the gait pattern was modified. Indeed, the AP MoS (*p* = 0.002), the AP BoS (*p* = 0.002), and the ML BoS (*p* = 0.003) showed a significant main effect of the session. Modifications in the gait pattern were accounted for by a greater AP BoS and AP MoS and a reduced ML BoS. Post-hoc analysis revealed that both groups increased the AP BoS and AP MoS at EPT (AP MoS: *p* = 0.002; AP BoS: *p* = 0.009). At LPT, the AP MoS was still higher with respect to BL (*p* = 0.023) while the AP BoS did not show significant differences (*p* = 0.082) compared to BL. Both groups reduced their ML BoS at LPT (*p* = 0.002) compared to BL. Also at EPT participant showed a trend of reduced ML BoS, but values did not reach statistical significance (*p* = 0.054). Despite narrowing of step width in all participants, i.e., lower ML BoS, the ML MoS did not show significant modifications over time (*p* = 0.239).

### Training Session

**Figure 4:**
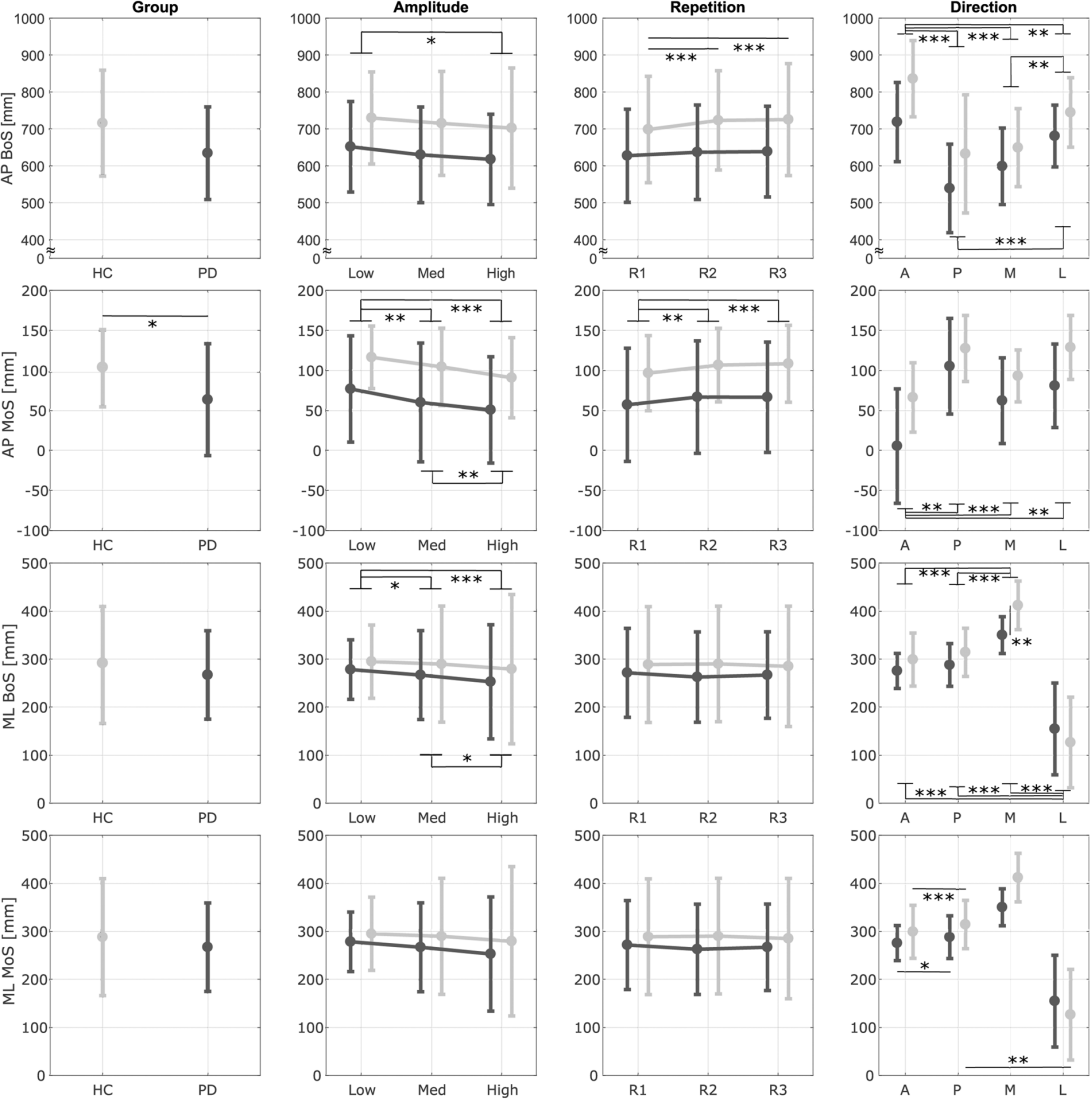
Results - Training Session. Antero-Posterior (AP) and Medio-Lateral (ML) Margin of Stability (MoS) and Base of Support (BoS)at Compensatory Heel Strike (CHS) for each group (Parkinson’s Disease - PD and Healthy Controls - HC), amplitude (Low, Medium, High), direction (Anterior - A, Posterior - P, Medial - M, Lateral - L) and repetition (R1-R3). Dark and light gray lines represent the PD and the HC groups, respectively. Bars refer to standard deviations. *Significant effect of the pairwise comparisons with Bonferroni’s correction (*p < 0.05, **p < 0.01, ***p < 0.001).

Figure 4 reports the results from the training session. Results showed that PD patients were less stable than age-matched controls in reaction to unexpected perturbations, independent of the direction, amplitude or repetition of the perturbations. The AP MoS showed a significant main effect of the group (*p* = 0.034) such that PD patients were characterized by lower values. Also the AP BoS showed a tendency to be lower in the PD group, but it did not reach the prescribed significance level of 0.050 (*p* = 0.087).

All outcome variables showed a significant main effect of amplitude (AP BoS: *p* = 0.002, AP MoS: *p* < 0.001, ML BoS: *p* < 0.001, ML MoS: *p* = 0.033). As expected, all outcomes were lower for higher amplitudes of the perturbations. Pairwise comparisons revealed that both groups showed significant differences between (i) Low and High amplitudes in the AP BoS (*p* = 0.014), AP MoS (*p* < 0.001) and ML BoS (*p* < 0.001); (ii) Low and Medium amplitudes in the AP MoS (*p* = 0.006) and ML BoS (*p* = 0.014); and (iii) Medium and High amplitudes in the AP MoS (*p* = 0.004) and ML BoS (*p* = 0.038). The AP MoS and the AP BoS showed a significant main effect of repetition (AP BoS: *p* = 0.020, AP MoS: *p* < 0.001). The multivariate test indicated a significant interaction effect between group and repetition for the AP BoS (*p* = 0.029). As expected, all outcomes were higher for subsequent repetitions of the perturbations. Pairwise comparisons revealed that both groups showed significant differences in the AP MoS between R1-R2 (*p* = 0.002) and R1-R3 (*p* < 0.001) but only the HC group showed significant differences in the AP BoS between R1-R2 (*p* < 0.001) and R1-R3 (*p* < 0.001). All outcome measures showed a significant main effect of direction (*p* < 0.001). The ML BoS and ML MoS showed significant interaction between group and direction (ML BoS: *p* = 0.001; ML MoS: *p* = 0.007). The AP BoS was (i) the largest for anterior perturbations (*p* < 0.006) and (ii) larger during lateral perturbations than posterior or medial ones (*p* < 0.001). The AP MoS was the smallest for anterior perturbations (*p* < 0.005). Both groups were characterized by higher ML MoS during posterior perturbations than anterior ones (HC group: *p* < 0.001, PD group: *p* = 0.039) but only the HC group showed higher ML MoS during posterior perturbations than lateral ones (*p* = 0.001). For both groups, the ML BoS was significantly wider during medial perturbations (*p* < 0.001) and narrowed during lateral perturbations (*p* < 0.001). However, the two groups showed significant differences for medial perturbations (*p* = 0.001) such that HCs were characterized by a wider step width than PD patients.

### Test Session

**Figure 5:**
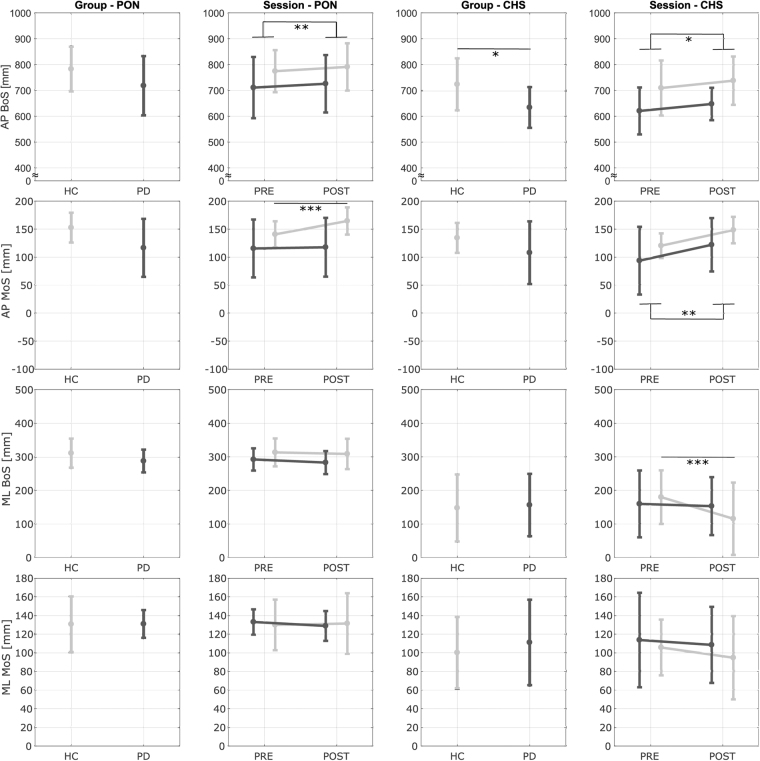
Results - Test Session. Antero-Posterior (AP) and Medio-Lateral (ML) Margin of Stability (MoS) and Base of Support (BoS) at Perturbation Onset (PON) and Compensatory Heel Strike (CHS) for each group (Parkinson’s Disease - PD and Healthy Controls - HC) and session (PRE and POST). Dark and light gray lines represent the PD and the HC groups, respectively. Bars refer to standard deviations. *Significant effect of the pairwise comparisons with Bonferroni’s correction (*p < 0.05, **p < 0.01, ***p < 0.001).

Figure 5 reports the results from the test sessions. None of the outcome measures showed a significant effect of repetition at PON or CHS (*p* > 0.085). At PON, the AP BoS (*p* = 0.007) showed a significant main effect of the session: all participants were characterized by an increased AP BoS at POST. The AP MoS showed a significant main effect of the session (*p* < 0.001) and interaction between session and group (*p* = 0.001). Pairwise comparisons revealed that only the HCs increased the AP MoS from PRE to POST (*p* < 0.001) such that they were characterized by an higher AP MoS at POST with respect to the PD group (*p* = 0.027). The ML BoS showed a trend to decrease from PRE to POST, but values did not reach statistical significance (*p* = 0.070).

At CHS, the PD group was characterized by a significantly shorter AP BoS (*p* = 0.032). Also the AP MoS showed a trend to be lower in the PD group, but values did not reach statistical significance (*p* = 0.092). Both the AP BoS (*p* = 0.034) and the AP MoS (*p* = 0.002) exhibited a significant main effect of the session. Both groups showed a larger AP BoS and AP MoS at POST than PRE.. The ML BoS presented a significant main effect of the session (*p* = 0.001) and interaction effect between session and group (*p* = 0.006). The pairwise comparisons revealed that only the HCs decreased the ML BoS at POST (*p* < 0.001).

## Discussion

The aim of this study was to investigate to what extent PD affects the ability to walk, respond to balance perturbations, and produce short-term adaptations to improve compensatory reactions and control of unperturbed walking balance. At present, only a few studies have addressed questions related to perturbed balance during walking in subjects with PD^44–47^. Still, it is unclear if Parkinson’s disease diminishes the ability to react and adapt to walking perturbations. It has been shown that PD affects the ability to integrate a reactive balance-correcting response during sideways perturbations while steadily walking^44^ but not during forward perturbations while switching from locomotion to gait termination^45^. In both cases, patients were able to implement proactive and reactive adaptions to improve their response^44,46^.

This study highlighted that, during unperturbed treadmill walking, patients with PD were characterized by a lower stability in the AP direction compared to HCs (Fig. 3). This is consistent with their typical stooped posture, shorter step length, and reduced ability to control the forward acceleration of the CoM^23,45^. This is the first study that analyzed the effects of multidirectional perturbations of different amplitudes delivered while walking in PD. Independent of the direction, amplitude, and repetition of the perturbations, PD patients were always characterized by a lower AP MoS at their first compensatory heel strike (Fig. 4). This is a sign of increased risk of falling and has been shown in patients with several neurological impairments when reacting to unexpected perturbations^48,49^. As expected, the recovery reaction was dependent on the direction and amplitude of the perturbations (Fig. 4)^35,36,38^. However, the ability to adapt to multiple perturbations or to modify the response to disturbances of different amplitudes or directions was not particularly affected by PD. The only differences were a reduced ability in PD to: (i) increase the AP BoS after the exposure to multiple perturbations of the same type; (ii) modulate the ML MoS in response to different direction of the perturbations; and (iii) react to medial perturbations with a large compensatory step width.

Results also showed that participants with PD were able to integrate and adapt reactive but not proactive behaviours. Indeed, only the HC group significantly increased their pre-perturbation stability at TEST POST (Fig. 5).

While the motor output associated with human walking has been largely associated with functions of spinal centers, both supraspinal and afferent inputs contribute considerably to the locomotor output^24^. Afferent inputs are critical for adapting the locomotor pattern, in a phase-appropriate manner, to unexpected perturbations^50^. Furthermore, the basal ganglia and the supplementary motor areas are important pathways for the modulation of locomotion^24^. The striatum, the cerebellum and frontal lobe are involved in motor learning and adaptation as well as online modification of movements like those seen during anticipation of a perturbation^46^. On the other hand, parkinsonian gait is characterized by neurotransmitter imbalances in the basal ganglia and under-activation of the medial frontal area, the right precuneus, and left cerebellar hemisphere^24^ and these are likely to be the cause for the inability of PD patients to modify gait pattern in anticipation of an upcoming perturbation.

Interestingly, patients with PD were able to adapt just as HCs their reactive strategies such that both groups increased post-perturbation stability at the end of the training. The similarity between groups suggests that most of the reactive adaptations were produced through modifications via extra-striatal sources. Presumably, given the specific neuromuscular skills and the fast nature of compensatory reaction, the adaptation of the reactive responses is mainly encrypted in lower parts of the central nervous system.

The exposure to repeated perturbations during training produced acute short-term aftereffects such that when cables were removed, the control of gait stability was modified during the 5 minutes of treadmill walking post-training (Fig. 3). Participants adjusted their gait pattern during unperturbed walking and did so even though they were aware that no perturbations would occur. This is of substantial importance if we want to induce generalized improvements in dynamic stability during unperturbed walking in everyday situations. Modifications of the gait pattern were accounted for by a greater AP BoS and MoS and a reduced ML BoS. Even though all participants narrowed their step width (i.e., lower ML BoS), the ML MoS did not show significant modifications. These results are in accordance with our previous studies with healthy young subjects^29^ and were effective to temporarily reduce the effect of PD on the gait pattern in the AP direction while walking on a treadmill. Indeed, patients with PD were characterized by a lower stability in the forward direction and a trend of reduced step length at BL (Fig. 3). Due to the training, PD patients temporarily increased step length and forward stability such that post-training values were closer to the ones showed by HCs at BL. In the ML direction, even if no differences were found between the two groups, a reduction in step width may be self-defeating in PD given that the gait pattern is generally characterized by lower values. However, we previously showed how the modification of the gait pattern is dependent on the direction of perturbations experienced during the training^29^. Healthy participants do not modify step width during unperturbed walking post-training if only perturbed in the AP direction. Future studies may have to involve only AP perturbations in patients with PD in order to avoid undesirable adaptations of the gait pattern.

Participants were tested in their clinically “on” state. Dopaminergic medications have shown mixed effects in previous studies that analyzed balance perturbation while standing. The postural response changes range from being absent^12,51–54^, worse^15^, or poor^5,55–57^. Similarly, some studies have reported positive^58^ and negative^52^ effects on the adaptation of the response. Reduced gait speed and shortened step length tend to be more accentuated in the “off phase”^25^. The fact that differences were more accentuated for the MoS than the BoS may indicate that the MoS is a measure that better relates to falls risk. Future studies need to further investigate how medication affects the reaction to perturbations delivered while walking.

A strength of this study is that walking speed was the same in the two groups. Spatio-temporal and stability parameters are altered as a function of gait speed both during normal walking and in the reaction to balance perturbations^59^. Accordingly, we were able to better isolate the effect of PD on the worsening of the motor response from alterations in the initial speed.

Our PD patients were in mild disease stages (H&Y 1–2). It has been suggested that the most useful gait biomarkers in PD for the development of potential neuroprotective interventions are those sensitive to early disease changes^60^. It is very encouraging that, despite patients were on medication, at early H&Y stages, and walking at the same speed as HCs, we were able to detect abnormalities that affect the ability to control the AP MoS both during unperturbed treadmill walking and in the reaction to unexpected perturbations in PD. Note, our sample size was relatively small and this may have affected the strength of our conclusions. Further studies involving a large number and a wider range of severity of the subjects are necessary.

With the type of perturbations used in the present study, we did not want to replicate mechanisms of common causes of falls (e.g., tripping and slipping). It is unclear if the effects of perturbation-based balance training are specific to the nature of the perturbations experienced^18^. Thus, it remains uncertain whether improved reaction to waist-pull perturbations would also apply to other disturbances. Even though the perturbations used in the test sessions were delivered in different directions to the ones experienced during the training, they were still very similar as their points of application on the human body were the same. Thus, observed proactive and reactive adjustments may be dependent on the specific type of the perturbations experienced and may not show transfer to other forms of perturbations in everyday situations. Future studies will further investigate this issue.

The present study only looked at very acute effects (unperturbed walking post-training was analyzed only for five minutes of treadmill walking). We did not expect the short intervention to have lasting effects that can be transferred to overground walking. Despite this, a recent study has shown how a single session of treadmill gait training with three-dimensional tilting perturbations of the surface is more effective than unperturbed walking in increasing overground walking speed^47^. Thus, we believe that increased stability may be transferable to overground gait but future studies have to confirm this.

Gait and balance disorders are one of the principal causes of falls and fall-related injuries^2–4^. Up to 70% of advanced PD patients fall at least once a year and two-thirds of these patients fall recurrently^2^. These rates are twice as high compared to healthy old subjects^61^. The consequences of such an event are traumatic, both physically and psychologically, leading to a decreased quality of life. Given the increase in older population and a higher incidence of PD among the elderly^62^, fall-prevention programs are becoming a major health challenge, not only to reduce costs, but also to mitigate personal and societal impacts.

A single session of the proposed therapy induced acute effects able to improve gait function and reactive reactions to unexpected perturbations for a short amount of time. This finding is encouraging for therapeutic interventions since a greater stability while walking and reacting to unexpected perturbations is associated with a reduced risk of falling. Future studies need to examine whether a multi-session training will demonstrate lasting effects on walking balance, accompanied by improved functional levels and a reduction in the number of falls during long-term follow-up assessments.
